# The role of ICT in supporting disruptive innovation: a multi-site qualitative study of nurse practitioners in emergency departments

**DOI:** 10.1186/1472-6947-12-27

**Published:** 2012-04-02

**Authors:** Julie Li, Johanna Westbrook, Joanne Callen, Andrew Georgiou

**Affiliations:** 1 Centre for Health Systems and Safety Research, Australian Institute of Health Innovation, Faculty of Medicine, University of New South Wales, NSW 2052, Australia

## Abstract

**Background:**

The disruptive potential of the Nurse Practitioner (NP) is evident in their ability to offer services traditionally provided by primary care practitioners and their provision of a health promotion model of care in response to changing health trends. No study has qualitatively investigated the role of the Emergency NP in Australia, nor the impact of Information and Communication Technology (ICT) on this disruptive workforce innovation. This study aimed to investigate ways in which Nurse Practitioners (NP) have incorporated the use of ICT as a mechanism to support their new clinical role within Emergency Departments.

**Methods:**

A cross-sectional qualitative study was undertaken in the Emergency Departments (EDs) of two large Australian metropolitan public teaching hospitals. Semi-structured, in-depth interviews were conducted with five nurse practitioners, four senior physicians and five senior nurses. Transcribed interviews were analysed using a grounded theory approach to develop themes in relation to the conceptualisation of the ED nurse practitioner role and the influences of ICT upon the role. Member checking of results was achieved by revisiting the sites to clarify findings with participants and further explore emergent themes.

**Results:**

The role of the ENP was distinguished from those of Emergency nurses and physicians by two elements: advanced practice and holistic care, respectively. ICT supported the advanced practice dimension of the NP role in two ways: availability and completeness of electronic patient information enhanced timeliness and quality of diagnostic and therapeutic decision-making, expediting patient access to appropriate care. The ubiquity of patient data sourced from a central database supported and improved quality of communication between health professionals within and across sites, with wider diffusion of the Electronic Medical Record holding the potential to further facilitate team-based, holistic care.

**Conclusions:**

ICT is a facilitator through which the disruptive impact of NPs can be extended. However, integration of ICT into work practices without detracting from provider-patient interaction is crucial to ensure utilisation of such interventions and realisation of potential benefits.

## Background

The concept of "disruptive" innovations arose over fifteen years ago to describe new technologies or interventions which create significant impacts on a commercial market [1]. In contrast to "sustaining" technologies, which provide incremental improvements to the performance and outcomes of existing technologies, disruptive innovations replace existing processes and eventually change the face of the market in unexpected ways. The progressive introduction of Information and Communication Technologies (ICT) in the healthcare system has been promoted for its potential to improve the quality and efficiency of care delivery through the automation of manual processes. However, it has been argued [2] that ICT provides the opportunity to revolutionise the healthcare system by enabling innovations in clinical work practice and changes to professional roles and responsibilities resulting in new models of care delivery. This includes the use of technologies, such as electronic decision support systems and online reference evidence sources [3-6] to expand nurses' roles to encompass tasks traditionally viewed as physician responsibilities supported by technology.

Emerging studies investigating the use of ICT by nurses in hospital settings have found that the increased volume and ease of access to patient and knowledge-based information has allowed registered nurses to extend and utilise their expertise to play a larger and more autonomous role in patient care [7,8]. In addition, telemedicine interventions address gaps in care delivery in rural areas by enabling remote management of patients by traditionally under-qualified staff with support from electronic decision support software [9]. In this way ICT may be viewed as a disruptive technology within the health system. However research examining the ways in which health professionals embrace technologies to create transformative change in health care delivery remain limited. Newly created professional roles, designed to cater for specific health system needs, may also be viewed as potentially disruptive technologies. A case in point is Nurse Practitioners (NPs). The disruptive potential of NPs as a new professional grouping lies in their significantly extended practice and impact within primary care [10], especially the operation of nurse-led clinics in the absence of GPs [11-13]. Further, NPs deliver an alternative model of care, emphasising a holistic and preventative healthcare paradigm which extends beyond a disease focus to encompass the psychosocial aspects of a patients whilst emphasising health education, self care and long term health maintenance [14-16].

The NP model of practice was introduced in Australia over ten years ago as one strategy to alleviate the maldistribution of General Practitioners (GPs) and the rising costs of chronic health management [17]. Senior nurses who have completed a University awarded Masters degree in Nurse Practitioner studies, NPs manage uncomplicated patient episodes autonomously and are authorised to undertake all investigative, prescriptive, and care coordination activities relevant to clinical cases which fall within a defined scope of practice [18]. Despite the objective of responding to the considerable shortage of primary healthcare practitioners, especially in rural areas, a national census of nurse practitioners by Gardiner and colleagues [19] found that NPs were most frequency employed in emergency medicine accounting for 27% of 145 survey respondents. Despite this, there has been little research investigating the role of NPs in emergency departments and the ways in which their role facilitates service delivery. Emergency departments' use of ICT has expanded rapidly and many departments are highly dependent upon clinical information systems to deliver efficient and high quality services. Investigation of the ways in which such technologies support the role of NP has been absent from the literature, despite ICT being a potentially powerful tool by which to support the transformative role of NPs in the health system. This study aimed to investigate the ways in which Nurse Practitioners (NPs) have incorporated the use of ICT as a mechanism to support their new clinical role within emergency departments.

## Methods

### Design and setting

A qualitative study using in-depth semi-structured interviews of clinicians from Emergency Departments in two large Australian metropolitan public teaching hospitals was undertaken. The hospitals were located within the same geographic Area Health Service and used the same centrally administered clinical information system. Qualitative research methods are effective for in-depth explorations of clinician perceptions regarding roles and clinical work practice [20]. Ethics approval for the study was granted by the relevant Area Health Service Research Ethics Committee and Human Research Ethics Committees of both hospitals and the University of New South Wales.

### Clinical information systems

Electronic clinical information systems in use at the hospitals were components of the commercially available Cerner Millenium suite of software solutions developed in the United States. The ED information system, Cerner FirstNet, monitors patient volumes and tracks patient movement and clinical activities within the department whilst providing an interface through which the hospitals' integrated clinical patient management system is accessed. Cerner PowerChart is a patient data repository comprised of many components of an Electronic Medical Record (EMR), accessible through FirstNet. It provides a Provider Order Entry functionality for orders such as pathology, medical imaging, diets, and transport requests. Also available electronically were diagnostic test results, test order status and discharge summaries from previous ED and inpatient admissions across the Area Health Service. PowerChart also contained various documentation templates including mandatory reporting forms, initial care plan template and discharge summary documentation. Diagnostic images were available electronically through Picture Archiving and Communication Systems (PACS). Knowledge-bases were accessible through the Clinical Information Access Project (CIAP) accessible via the hospital's intranet or via hyperlinks from Powerchart and FirstNet. Paper-based hospital medical records were used by doctors and nurses in the ED concurrently with the EMR and contained information such as vital signs, medications, and progress notes.

### Participant selection and sampling

Selection of sites was based on ED physicians' and nurses' use of the mandatory EMR system, high patient throughput and the heterogeneous mix of clinical presentations. Fourteen interviews were undertaken with five nurse practitioners, four senior physicians (two ED Directors and two senior staff specialists) and five senior nurses (three Nurse Unit Managers, an Advanced Clinical Practice Nurse and a senior Registered nurse) between July 2010 and January 2011. All nurse practitioners (n = 5) employed at the two EDs were interviewed whilst doctors and nurses were purposively selected based on seniority of role and length of employment at the sites to ensure knowledge of the development of the nurse practitioner role onsite and reasonable levels of interaction with nurse practitioners during clinical work. The Nurse Practitioner role had been introduced at both hospitals approximately four years prior to data collection. A document outlining the study, its voluntary nature, confidentiality of results and participants, and a consent form were witnessed and signed by participants following the opportunity to ask questions related to the study. Participants were recruited via email and approached personally in the department when email contact failed. Interviews with senior doctors and nurses regarding their understanding of the nurse practitioner role were triangulated with data from nurse practitioners who were further asked to express their perceptions of the impact of ICT on their role and work. Interview questions were informed by the relevant literature, a pilot interview with one NP participant and an observation of two nurse practitioners at one site prior to data collection, following which questions were revised. Table 1 displays interview questions for participant groups. This study reports findings specific to clinicians' understanding of the NP role and the NPs' perceptions of the impact of ICT in shaping their roles. Characteristics of the study sites and participants are summarised in Table 2.

**Table 1:** Lead questions guiding semi-structured interviews with Emergency Department physicians, nurses and NPs

Questions for Nurse Practitioners	Questions for Senior Physicians and Nurses
1. How would you describe your role?	1. What is your understanding of the role of the NP?
2. How is the role of a Nurse Practitioner (NP) different from that of doctors and other nurses?	2. What do you perceive is the impact of the NP role on the delivery of care?
3. Do you think that the roles of existing Emergency Department (ED) clinicians have changed as a result of the NP role?	3. How do you think the role of the NP influences the roles of doctors and nurses?
4. Do you think the roles of NPs would differ at other sites? - Why do you think they would differ? (e.g. due to ICT, casemix, culture etc.) - What are the mechanisms which determine the role of the NP?	4. What are the mechanisms that determine the role of the NP?
5. What clinical information systems do you use in your day-to-day work? - Which aspects/functions specifically?	5. What is your view on the overlap between NP roles and those of doctors?
6. Do you feel that ICT supports your work as a Nurse Practitioner? - How would you envisage your role in the complete absence of ICT? Would it be as expansive as it is now? Would duties be performed in the same way?	
7. How do you see the future of the NP role in EDs as ICT continues to evolve? - do you envisage taking on new responsibilities as the system becomes more developed?	
8. What is your view on the overlap between NP roles and those of doctors?	

**Table 2:** Characteristics of study sites and participants

Characteristics*	Hospital Site	
	
	A	B
Hospital Beds	445	758

Annual Discharges	29,939	83,898

Annual ED Attendances	51,105	61,939

Annual ED Discharges	40,323	40,713

**ED Interviews**		

Sample	4 Nurse Practitioners;	1 Nurse Practitioner;
	ED director;	ED director;
	1 senior staff specialist;	1 senior staff specialist;
	1 Nurse Unit Manager;	2 Nurse Unit Managers;
	1 Advanced Clinical Practice Nurse	1 senior registered nurse

* Hospital statistics over year 2009/2010

### Data analysis

A grounded theory approach using thematic content analysis [21] was employed to derive themes in relation to the conceptualisation of the ED nurse practitioner role and the perceived influences of ICT upon the role. Interview transcripts were analysed inductively over a multi-phase process. Interview questions were employed in the initial stage of analysis to form broad, macro-codes and a basic taxonomy under which open-coded content could be classified. Portions of transcribed interview text relating to each question were analysed by one researcher (JL) to develop lists of specific codes representing the various attributes and dimensions of each macro-code. Transcript extracts demonstrating a code was then matched with that code. Electronic management and coding of data using NVivo (v.8) software ensured automatic collation of all data extracts within each code. Coding reached theoretical saturation when no new themes emerged. Axial coding of related data extracts within and across categories was undertaken in a second phase to identify meaningful relationships between codes and higher level, recurring themes. Revision and finalisation of themes was achieved via triangulation and consensus between all researchers (JL, JW, JC, AG). Member checking of results occurred through follow up interviews with relevant participants to clarify findings with participants and further explore emergent themes.

## Results

### Role of the nurse practitioner

#### Advanced practice

Views about the role of the Nurse Practitioner were explored and triangulated between all NPs and physician and nurse participants across sites. All participants acknowledged the advanced practice aspect of the NP role, understood broadly as an extension of the skills, experience, and knowledge demonstrated by Registered Nurses. Respondents reported that the expanded role of NPs involved exercising appropriate decision-making and care coordination tasks to manage entire patient episodes, including the assessment, diagnosis, treatment, discharge planning and referral, of patients who fell within a defined scope of practice; represented generally by sub-acute presentations such as uncomplicated injury, trauma and musculoskeletal injuries, urinary symptoms, skin infections and ear, nose and throat complaints. Participants understood that NPs were authorised to order all investigative procedures relevant to their cases and prescribe from a formulary customised to their scopes of practice. Respondents acknowledged that NPs practised autonomously in the management of their own patients, collaborating with senior physicians on complex clinical cases.

NPs also highlighted non-clinical facets to their role, citing academic activities such as research and contributing to the scientific literature in areas of clinical interest and expertise, staff education during formal internal training sessions such as Grand Rounds, and clinical leadership and supervision of other nurses aspiring to advanced practice. Whilst the clinical domain of the role was fulfilled in abundance, NPs at one site felt that non-clinical elements lacked emphasis in routine practice and anticipated greater recognition as the NP role continued to develop.

#### Holistic care

The purpose of the NP role was largely perceived by physicians as the alleviation of their sub-acute workload and expediting the treatment of patients of lower acuity. NPs and senior nurses reported a holistic aspect which defined their practice and which they believed distinguished their role from that of a physician. Holistic care was understood to be an expansion of the clinical focus beyond the disease to the whole person; consideration of the physical as well as the psychosocial needs of a patient. NPs believed they held a deeper insight into other health professions and engaged allied health professionals more frequently in the provision of a comprehensive strategy of care. Senior nurses felt that NPs delivered care with the medical expertise of a physician (within their defined practice areas) and the "caring" of a nurse. NPs and senior nurses agreed that NPs were more likely to spend more time with patients for the purposes of education and health promotion. One senior nurse felt that having a sole practitioner for entire patient episodes resulted in greater continuity of care and a greater client focus.

### Nurse practitioners' use of ICT

Nurse practitioners were asked to describe their use of the available ICT in clinical work. All NP respondents agreed that their use of the Cerner systems replicated that of physicians. NPs utilised FirstNet to review triage notes prior to physical examination and electronically allocated patients to their care. Previous notes in PowerChart were also accessible before and during patient encounter. The provider order entry functionality in PowerChart was accessed to request pathology and medical imaging tests, and NPs reported viewing radiological results through PACS and pathology results via PowerChart. All NPs testified to the value of knowledge-bases and cited regular reference to electronic medical and drug textbooks, therapeutic guidelines and clinical decision tools to support and verify diagnostic and therapeutic decisions throughout their practice.

Documentation practices differed between NPs. Because PowerChart was not used to document progress notes or for medication documentation, some respondents reported concurrent documentation in both paper and EMRs whilst others used the care plan and the electronic discharge summary templates to record entire care episodes. All electronic notes were printed and added to the paper medical record. NPs discharged patients from the department electronically using FirstNet following conclusion of the episode of care.

### Impact of ICT on role

#### Factors shaping the nurse practitioner role

Nurse Practitioners and senior physicians and nurses were asked to reflect upon factors which shaped the NP scope of practice. Directors at both EDs reported the significant role that existing NP models in rural Australia and the UK played in the development and implementation of the NP role in their hospitals. Senior staff specialists identified departmental needs and patient presentation trends such as the increased numbers of less serious cases as important in the shaping of the role. Nurse Unit Managers and senior nurses felt that the evolution in the division of medical labour, advancements in medical technology, as well as the acceptance by departmental and ancillary staff as important in the successful implementation of the NP role. NPs added that the confidence of individual NPs shaped the extent and nature of their practice in the department.

#### Enhanced decision making

Whilst ICT was not immediately recognised as a direct influence on the NP role, when asked to expand on the integration of ICT in their work, NPs revealed that the accessibility of existing patient information significantly facilitated their clinical decision making processes and this was a central component of advanced practice. Unlike paper medical records which required delivery from local or remote medical record departments and potentially taking 1 to 24 hours, EMRs were accessible in real time. This was considered especially important as many NP-managed encounters may end prior to the arrival of the paper record. Electronic availability of images through the PACS system immediately upon completion of the investigation expedited access to radiology results by negating the need to collect physical films from the radiology department or awaiting its transport by ward staff people. NPs perceived that the real time availability of relevant clinical information expedited their decision making processes and shortened the gap between initial patient examination and subsequent access to care.

NPs reported that access to previous records resulted in a higher quality of decision making and improved patient safety. Integration of patient databases across hospitals in the Area meant that patient information across sites was available and thus decisions would be based upon a more complete body of information. This was extremely helpful for patients who may have rarely or never presented to the site. Access to complete clinical patient information was also important in the management of older patients with extensive medical histories and those from culturally and linguistically diverse backgrounds who encounter difficulties recounting their clinical history.

Another advantage of electronic information was its ubiquity and permanence compared to paper records and radiographic films which may become lost or misplaced. Organisation and display of both recent and previous patient radiographic and pathology results are presented in one view on the computer screen, and may be graphed and are customisable, facilitating comparison with previous results and trend-spotting. One NP recounted how she was initially alarmed by an ostensibly abnormal creatinine result but was informed by previous results that the value was a normal baseline for the patient. Electronic patient data may also be accessed by multiple parties at multiple sites simultaneously. One NP stated that the ability to confer with external specialists whilst both referring to common information improved the quality of communication during consultations and was extremely beneficial to practice.

#### Improvements to system

NPs were invited to suggest improvements to the existing ICT to further support their role. Whilst opinions of existing systems were generally favourable, a prominent point raised was the relative scarcity of computers in work areas which rendered access and use problematic at times. Currently, NPs would coordinate work around the availability of computers by deferring electronic tasks for manual procedures until a computer was vacated. NPs also requested increased expansion of the integrated Cerner database network to extend beyond the one Area Health Service to encompass the entire country, and to allow input from all healthcare settings and providers, such as primary healthcare practitioners to improve completeness of patient information and facilitate seamless patient transitions across healthcare providers.

NPs requested further expansion and sophistication of the system to include functionalities such as progress note documentation and medication management to create a single, complete EMR accessible during encounters; despite the timeliness of electronic data, paper medical records remain the more comprehensive for patient encounters at the site, containing paper printouts of electronic entries such as discharge summaries and transcriptions of relevant patient data such as test results. One NP also recounted her experiences at other sites and the benefits of an electronic medication module. Its ability to display extensive medication histories within the one, scrollable view reduced the need to locate multiple paper charts and duplicate recordings of the same medication on separate charts as paper charts reach capacity and patients are transferred across departments. The ability to integrate decision support including links to therapeutic guidelines and alerts to drug interactions was further envisioned to reduce error and support advanced practice. Finally, integration of digital images in the EMR for graphic monitoring of certain conditions, such as wounds, was suggested by another NP. Representative quotes of NPs' perceived impact of ICT on roles are presented in Table 3.

**Table 3:** Impact of ICT on NP role - representative quotes

Category	Quotation
Enhanced decision making	"when you think of something like [PowerChart] that's also helpful because you can electronically click back to someone's previous attendances which is usually very useful if they've represented with the same case or, gives you an idea if they're a regular attender here. You can look up their past blood results and do a comparison with how their bloods are today so, it's very useful. It definitely allows you to get a better picture as long as they've presented within [the Area Health Service] then you should have a lot more data on them than you would otherwise - otherwise you've got to ask a clerk to go and get their old notes and we might not have them and if they're offsite it could take 24 hours, so definitely expediates [sic] care, makes it faster and I think, probably gives you more information on which to base your decision making" [Site A, NP 3]
	"it's great when you have a patient coming back, that's been here a week ago or whatever, when I can access and all the notes are on the system. It's brilliant because it's so hard when they're not, because you have no idea. The patient tells you something and they're not quite sure" [Site A, NP 2]
	"viewing x-rays on the system, on the PACS system has a positive impact. Especially because I can talk to a registrar at [another site] or I can talk to a registrar here and he's looking at the same x-ray. That's really useful. So that impacts on my work. In a very positive sense that does" [Site A, NP 1]
	"I can see all previous x-rays and all of the pathology comes back onto the system so again I can see layers of pathology, or dates of different pathology: I can see your last ten blood tests and do comparisons" [Site A, NP 1]
	" I had a patient who came in with ischemic toe and his creatinine was a thousand...you see something as ischemic toe and then, looking at his previous blood results his creatinine was the same" [Site B NP]
	"there are certainly advantages. I can do patient tracking so I can see what patients I've got. You can't lose notes in [PowerChart] as we're forever losing patient notes here like the paper notes. They're always being lifted by somebody and taken somewhere" [Site A, NP 1]
	"I think now the systems in place you're not forever looking around for x-rays. You don't lose them" [Site B NP]

Improvements	"the only thing I find limiting is...a lot of the time you're struggling to get a computer and that's the frustration" [Site B, NP]
	"physical access to [a computer] is sometimes really difficult" [Site A NP 4]
	"Certainly more computers or PDAs or iPads or something like that. We've got COWs *[Computers On Wheels]*. They're okay but they're so big...imagine carrying one of those things everywhere. It's doable but anyway" [Site A, NP 1]
	"what it has the power to do is improve the care given to patients so if everywhere in [the state] was on the same system and if everybody including GPs, clinics, private or public, if everyone had access to the PACS system then we would no longer have to print disks off all the time for GPs so I think for patients it has the ability to make things completely seamless and so if they'd been to a GP, I might then be able to click on [PowerChart] and actually access their last GP notes which would be incredibly useful to me because the number of people that come in here that say "I normally go see my GP but now I'm here", then I'm like, you know I wonder what they've been saying to their GP, is it different, or does this leg look any different? How on earth would I know?... so for me it would allow me to probably make more accurate decisions, otherwise I'm just going by what the patient's told me and quite often it's just very subjective information isn't it?... it could vastly improve the care of patients" [Site A, NP 3]
	"it just needs to be expanded, it really needs to be expanded...we need to get things like medication charts, that's one thing. We really need to get medication charts on the system because that would wipe out so many errors - medication errors - if you have medication charts online. I worked with it before and it's brilliant because you can't have two medication charts and...it's all there. If it's signed for it's given, if it's not signed for it's not given and things like that it just, it's brilliant" [Site A, NP 2]

## Discussion

Senior doctors and nurses across both hospital EDs agreed on the broad advanced practice dimensions of the NP scope of practice and acknowledged the autonomous nature of NP practice which differentiated the role from that of other clinical nursing roles in the ED. Whilst not specifically acknowledged by senior physicians, NPs and senior nurses perceived that NPs considered the psychosocial needs of the patient in addition to the clinical, and embraced more readily a team-based approach to care delivery. In addition, NPs at one site felt the non-clinical aspects of their role were under-emphasised in practice and lacked recognition by other doctors and nurses.

### ICT as a facilitator of innovation

Many factors determined the NP role and scope of practice. While ICT was not initially identified as shaping the NP role, NPs agreed upon exploration of work practices that the integration of ICT into their work facilitated the advanced practice element of their role through contributing to the timely and quality of clinical decision making. The inability of participants to immediately conceptualise ICT use as separate from work processes indicates the extent to which NP have integrated technology into their nursing practice.

The accessibility and availability of electronic patient information through an integrated Area-wide database enhanced clinical decision making in two ways. Real-time access to requisite patient information was believed to expedite clinical decision making and patient access to appropriate care. Ubiquity of electronic patient information informed clinical decision making through a centralised patient database which sourced all hospitals in an Area. Although not greatly emphasised, one NP hinted at the potential of ICT to support a team-based, holistic model of care delivery through the ubiquitous nature of electronic information from a common patient database, facilitating information-sharing and communication between all health professionals across departments both within and across sites. The anticipated geographic expansion of the patient database and extended functionality of the EMR to reconcile the existing dichotomy of manual-electronic patient information repositories by a number of NPs is likely to see this benefit extended.

The reality of the NP role in reaching its disruptive potential is a contentious issue. International researchers have based the disruptive impact of NPs on their ability to complement physicians in care of subacute patients and their better accommodation of changing population health needs by espousing a preventative care paradigm. Because the NP role remains relatively new in Australian, the scarcity of NPs, their poorly defined and still-emerging roles, and existing healthcare regulations, professional standards and administrative procedures presently limit the impact of the NP beyond that of a sustaining innovation [22]. However, the findings from this early study convey the considerable potential of ICT in advancing the disruptive characteristics of the NP role.

Results from this study complement the existing literature in exploring the integration and use of ICT to support roles in differing settings. The time-pressed ED environment saw NPs from our study emphasising the accessibility of electronic patient notes over remotely-located paper notes in the facilitation of timely diagnostic and therapeutic decision making, a key component of advanced practice. While knowledge-databases and clinical decision making tools were regularly used and cited as useful by our NP participants, the utility of electronic decision support elements of ICT were more thoroughly explored among NPs in primary care [23,24] and critical care [25] settings where decision support software was perceived to support advanced practice and improve quality of care. Also raised as an area of interest in this study, support for electronic prescribing and medication management was acknowledged as an advantage by participants across a variety of care settings [26]. However, successful integration of ICT into clinical work practices emerged as a consistent prerequisite for ICT adoption before potential benefits can be realised [23-28]. Whilst NPs in this study cited the lack of computer terminals as a barrier to ICT use, awkward user interfaces, relative computer illiteracy and perceived disturbances to the patient encounter and nurse-patient rapport were additional factors reported as limiting utilisation of such interventions.

As the NP role continues to expand and proliferate in the Australia healthcare system, the increased diffusion of mobile technology such as Personal Digital Assistants (PDAs) will support extension of non-clinical dimensions of work which may see practitioners removed from traditional treatment areas where stationary devices are readily available [24,26]. Multimedia and telemedicine capabilities will facilitate communication with distant or offsite colleagues, supporting the holistic, team-based paradigm of care central to the advanced practice model of nursing.

### Limitations

Qualitative, exploratory studies looking at the use of ICT by NPs and their associated impact on role are scarce. Studies have sought to examine the relationship between ICT use and clinical indicator or outcome [29], quantitative, descriptive studies examining rates of use [30,31] and statistical correlations between user attributes and patterns of use [30]. Indicator data are valuable in providing a framework for evaluation, however they are limited in terms of explaining the nature of the relationship between variables or understanding the various contextual factors which often contribute to outcomes [20]. This study aimed to address this gap in providing in-depth insights into the influence and impact of ICT on facilitating the potentially disruptive role of NPs. The findings present new evidence about the ways ICT is being integrated and is helping to shape the role of NP, however, the study was conducted at an early stage of NP implementation across sites and findings are likely to vary as both role and systems become increasingly established.

## Conclusions

ICT facilitates the advanced practice of NPs through enhanced timeliness and quality of diagnostic and therapeutic decision making. The integration of patient data from all hospitals within an Area Health Service was judged to support and improve the quality of communication between health professionals within and across sites, with wider diffusion of the EMR holding the potential to further facilitate team-based, holistic care. Poised at the nexus of ICT use and NP practice is a new care paradigm set to transform the landscape of care provision. ICT is a facilitator through which the disruptive impact of NPs can be extended. However, integration of ICT into work practices without detraction from provider-patient interaction is integral to ICT adoption and realisation of potential benefits.

## Competing interests

The authors declare that they have no competing interests.

## Authors' contributions

All authors contributed to the conceptualisation and design of the study. JL undertook data collection, analysis, and drafting of the manuscript. JW, JC, and AG contributed to results interpretation and critical revision of the manuscript. All authors read and approved the final manuscript.

## Pre-publication history

The pre-publication history for this paper can be accessed here:

http://www.biomedcentral.com/1472-6947/12/27/prepub
